# Supplementary figures and images for: Comparison of two molecular barcodes for the study of equine strongylid communities with amplicon sequencing
Source: PeerJ. 2023 Apr 12;11:e15124. doi: 10.7717/peerj.15124 (PMC10105562; doi:10.7717/peerj.15124)

A

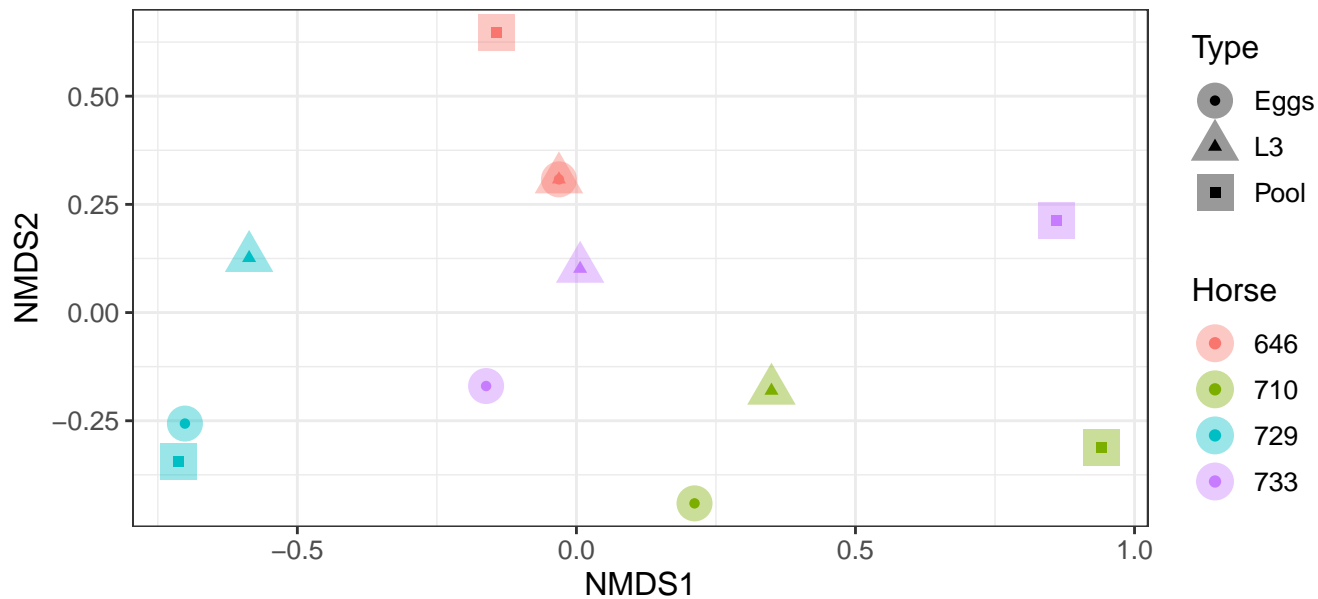

B

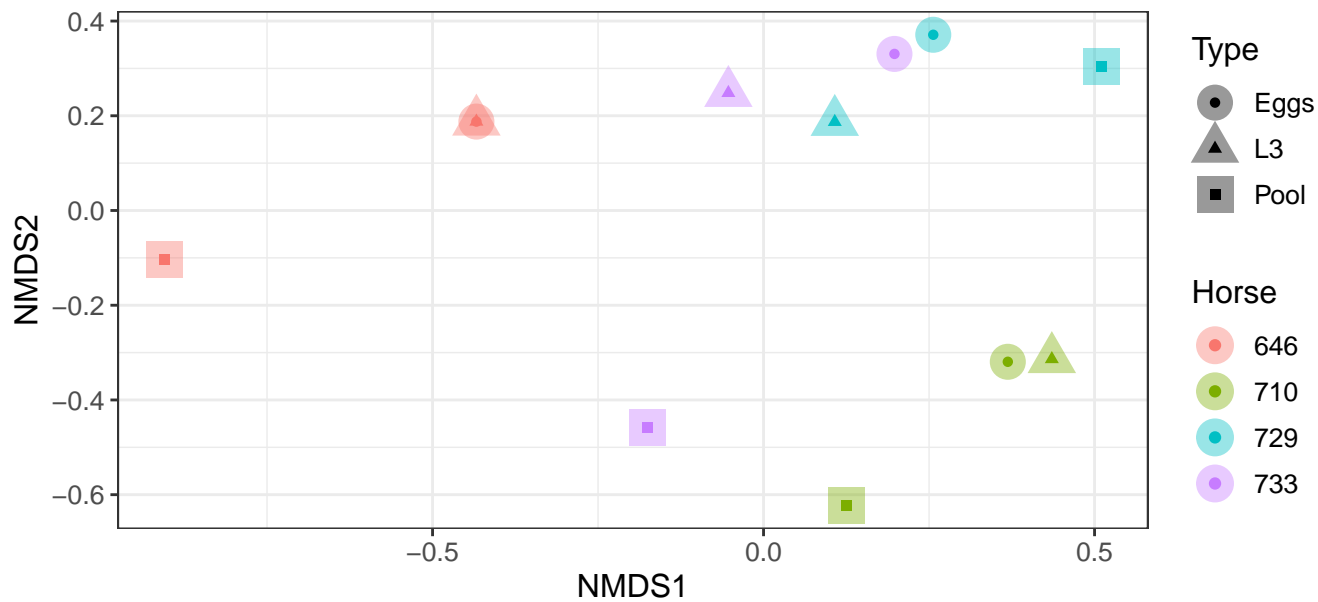

Supplement: Supplemental Information 7 [file peerj-11-15124-s007.pdf]
